# Travesty of Undetected Hypoparathyroidism: A Series of Missed Opportunities

**DOI:** 10.7759/cureus.40876

**Published:** 2023-06-23

**Authors:** Namitha Shaji, Ankit Tiwari, Penuboina Tejaswini, Nupur Goyal

**Affiliations:** 1 Department of Internal Medicine, All India Institute of Medical Sciences, Bhopal, Bhopal, IND; 2 Department of Cardiology, Postgraduate Institute of Medical Education and Research, Chandigarh, Chandigarh, IND

**Keywords:** hypocalcemia, bone marrow suppression, seizure, bilateral intracranial calcification, severe aplastic anemia, fahr´s syndrome, non surgical hypoparathyroidism

## Abstract

We describe a 20-year-old male with childhood-onset seizures and a prolonged history of anti-epileptic use. The cause of his seizures remained undetected until he reached the second decade of his life. Extensive intracranial calcifications on brain imaging helped us identify hypocalcemia as a cause of seizures. He had low calcium due to primary hypoparathyroidism. He also had severe aplastic anemia at this time. There were a series of missed opportunities in his history that could have prevented prolonged anti-epileptic use and probably preserved his marrow. This is an educational case for all physicians on how parathyroid abnormalities may get missed.

## Introduction

Parathyroid disorders have myriad presentations, and their diagnosis often requires a high index of suspicion. Hypoparathyroidism is an uncommon condition that leads to hypocalcemia. Clinical features of subtle hypocalcemia are non-specific and can be easily missed. To a pediatrician, hypocalcemia-induced seizures may be mistaken for idiopathic epilepsy [1]. Brain imaging, which reveals bilateral basal ganglia and cerebral calcification, could prompt a search for underlying calcium abnormalities and, hence, a diagnosis of primary hypoparathyroidism [2]. The presentation of either nephrocalcinosis, renal insufficiency, bilateral cataracts, or cardiac arrhythmias in childhood could also be a prompt to measure serum calcium levels. Our patient was from a rural background and had never been evaluated previously for the cause of his seizures. The delayed presentation and nature of the disease eventually led to an unfavorable outcome.

## Case presentation

A 20-year-old Indian male had been having generalised tonic-clonic seizures since the age of three. Since the onset of seizures, he has been on oral phenytoin. He was born at term via normal vaginal delivery, which was uneventful. His mother did not have any history of illnesses or drug intake in the antenatal period. There was no history of developmental delays. Initially, he had adequate seizure control, except during brief, intermittent periods of drug non-compliance. In view of seizure recurrence, two years later, oral clobazam was added to phenytoin. At 10 years of age, he developed blurred vision and was diagnosed as having bilateral cataracts. Bilateral intra-ocular lenses were implanted, and his vision improved. He continued taking his anti-epileptic drugs (AEDs). He dropped out of school a year later due to financial constraints and started helping his father at work. He was a teetotaler and had no history of illicit drug use. There was no significant family history. At age 18, his parents noticed a change in his speech. Initially, his speech became slow and slurred. Over the next year, his speech deteriorated further, as he was able to speak only a few disjointed words. His parents believed these symptoms to be due to an underlying seizure disorder and did not seek a specific evaluation. He, however, continued to receive his prescription refill. Two years later (two months before his presentation to us), he developed a tingling sensation around his lips. At this time, he was detected to have severe pancytopenia (haemoglobin [Hb], 3.9g/dl; 3210 leukocytes and 25,000 platelets per µl). He also had macrocytosis (mean corpuscular volume [MCV]: 120 fl), but there were no hyper-segmented neutrophils. His serum phenytoin levels were normal (17 mcg/mL). He received three units of packed red blood cells, and his anti-epileptic medication was changed to oral Lacosamide 200 mg once daily and oral Levetiracetam 750 mg twice daily. He was also advised to take oral vitamin B12 supplementation. Two months later, he had a generalised tonic-clonic seizure when he presented to us for the first time. This seizure lasted for five minutes, and then there was a 15-minute period of unconsciousness.

On presentation, he had significant pallor, two enlarged, firm, mobile lymph nodes (2 cm × 2 cm) in the right posterior triangle of the neck, and a male pattern of baldness. His vitals were stable, and his remaining general examination was unremarkable. We found his mini-mental status examination score to be 25/30 and his Montreal cognitive assessment score to be 26/30. His fluency and repetition of speech were affected, but his comprehension was intact. He had tongue fasciculations. He also had bilateral dysdiadochokinesia and past pointing. His gait was slow and broad-based. There were no tremors or abnormal movements. His Chvostek’s and Trousseau’s signs were negative.

**Figure 1:**
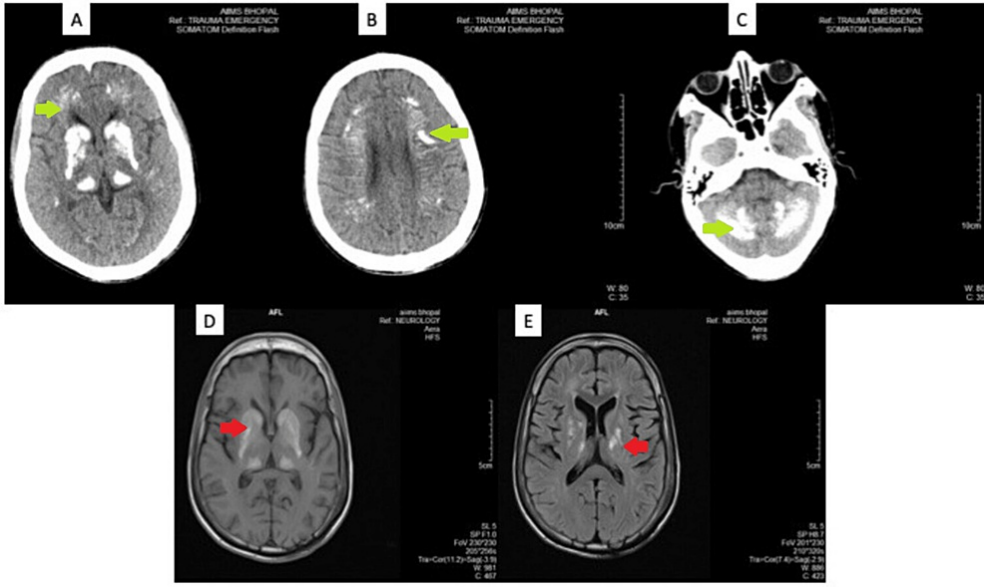
Non-contrast CT and MRI images showing symmetrical areas of bilateral intracranial calcification (A)-(C) are NCCT images showing symmetrical areas of calcification (green arrows) in bilateral basal ganglia and thalami, head of caudate nucleus, lentiform nucleus, and thalamus (A), periventricular white matter (B), and cerebellar hemispheres (C). (D) and (E) are corresponding MRI images showing symmetrical areas of altered signal intensity (red arrows) involving bilateral basal ganglia, thalami, periventricular and deep white matter, corona radiata, centrum semiovale (D). There also are multiple small confluent areas of gliosis in bilateral centrum semiovale, suggestive of old infarcts (E).

At the time of current admission, he had persistent pancytopenia (Hb 2.8 g/dl, 2742 leukocytes, and 20,000 platelets per µL). His macrocytosis had improved (MCV 103 fl). The peripheral smear showed marked anisocytosis. He did not have any azotemia (serum creatinine 0.66 mg/dL), and his serum sodium and potassium levels were normal. He had low total serum calcium (4.86 mg/dl [normal range: 8.5-10.5 mg/dl]). His serum bilirubin and liver enzymes were normal. His serum protein was 7.5 mg/dL, and albumin was 3.8 mg/dL. Due to the persistence of pancytopenia and the presence of cervical lymphadenopathy, we were initially worried about marrow-infiltrating haematological malignancies. Bone marrow aspiration and biopsy showed hypocellular marrow with a marked reduction of all cell lineages. There were no abnormal cells in the bone marrow. We obtained cytology of cervical lymph nodes, which revealed ill-formed epithelioid cell granulomas with a polymorphous population of lymphoid cells. Lymph node cytology was negative for acid-fast bacilli. The aspirate from the lymph node was negative for tuberculosis on the cartridge-based nucleic acid amplification test (CBNAAT). There were no additional thoracic, abdominal, or neck lesions on CT imaging. His hepatitis B, hepatitis C, and HIV screenings were negative. His iron, vitamin B12, and folate levels were within normal limits. Other than a long history of anti-epileptic drug intake, there was no other obvious cause for aplastic/hypoplastic anaemia. Cervical lymphadenopathy could also be attributed to chronic phenytoin use (Figure 1).

Seizures from early childhood and no prior imaging records prompted us to obtain a CT head as part of a screening investigation. His plain CT head was done, and we found bilateral extensive hyperdense areas (mean HU 139) in the basal ganglia, ganglia-capsular region, thalamus, and cerebellum. These symmetric calcifications were also corroborated on the MRI brain. These findings and the history of paediatric cataracts led us to a metabolic screen. He had hyperphosphatemia (6.52 mg/dl (normal range: 2.5-4.5 mg/dl)), hypercalciuria (400 mg in 24 hours [normal range: 100-250 mg/day]), and normal serum magnesium levels. His serum parathyroid levels were also low (1.7 pg/ml [normal range: 14-65 pg/ml]). These features were suggestive of primary hypoparathyroidism. His other endocrine hormone levels (thyroid hormone, serum follicle-stimulating hormone [FSH], luteinizing hormone [LH], and prolactin) and autoimmune profile (anti-thyroid peroxidase [TPO] antibodies and anti-neutrophil cytoplasmic antibodies [ANCA]) were normal.

During his hospital stay, we initiated him on injectable calcium gluconate for the initial three days, followed by oral calcium (2000 mg per day in divided doses) and oral calcitriol 0.25 µg once daily. He was also transfused with packed RBCs and platelets, and over the following week, his haemoglobin improved to 8.5 g/dl. As his serum calcium levels improved to 6.2 mg/dL, we tapered his anti-epileptics to oral Levetiracetam 500mg once daily over the next month. Oral hydrochlorothiazide was initiated at 12.5 mg once daily to reduce hypercalciuria. He was advised of bone marrow transplantation and was initiated on the bone marrow stimulant oral Stanazol 90 mg per day in divided doses. Over the next three months, he remained seizure-free, and his last recorded serum calcium was 6.32 mg/dL. His bone marrow, however, did not recover, and he required repeated blood transfusions. We could not get his bone marrow transplant done, as it was not available at our centre, and the patient’s family did not agree to shift him for specialised management. He died five months later, at his home, likely due to aplastic anaemia-related complications.

## Discussion

There were various missed opportunities in our case. His hypoparathyroidism could have been detected in childhood. First, recurrent seizures should have prompted metabolic screening and brain imaging. The recurrence of his seizures was attributed to medication non-adherence rather than an underlying metabolic cause. Second, the development of bilateral cataracts should have prompted metabolic and chromosomal screening. While he did not have a classical phenotype associated with major chromosomal defects, the subsequent development of bone marrow hypoplasia could have a genetic basis. Hypoparathyroidism was likely the underlying cause of his bilateral cataracts, which remained undetected for many years. Last, we do not have a definitive cause for his aplastic marrow. We believe it to be secondary to AEDs, but it is also possible that this was part of a syndromic presentation. Hypoparathyroid syndromes are rare and could have an underlying genetic basis. Certain mitochondrial DNA deletion syndromes (such as Pearson-Marrow-Pancreas syndrome) have co-existence of endocrine and haematological abnormalities, but these have a presentation in infancy, while our case was in the second decade of life [3].

The presence of bilateral basal ganglia and cerebral calcifications with neurological manifestations is termed Fahr’s disease. While named after a German pathologist, Karl Theodore Fahr, it is suggested that this nomenclature be avoided. There are numerous causes of diffuse cerebral calcifications, and Fahr may not be the first to describe them [4]. In our case, primary hypoparathyroidism explains such calcifications. The occurrence of diffuse calcifications also explains various neurological features such as dysarthria, cerebellar signs, and tongue fasciculations. The aetiology of co-existing aplastic anaemia and cervical lymphadenopathy remains speculative. We believe that the most likely culprit is phenytoin, which our patient has been taking for more than 15 years. While phenytoin-induced bone marrow aplasia is rare, it is well-described [5].

The pathogenesis behind the intracranial calcifications is an iron transport defect and the production of free radicals. These free radicles initially trigger intravascular calcifications and eventually extend to involve the neuron [2]. It has been reported that hyperphosphatemia has a significant correlation with vascular calcification as well. Human smooth muscle cells (HSMC) have been found to undergo mineralization in excess in an environment where there is an increase in extracellular inorganic phosphate levels, as the elevated phosphate level has been found to directly stimulate HSMC to undergo changes in its phenotype that favour calcification [6].

Bilateral extensive basal ganglia and cerebral calcifications have not only been described with hypoparathyroidism but also with hyperparathyroidism and pseudohypoparathyroidism [7]. Other secondary aetiologies include vitamin D disorders, mitochondrial myopathy, neurodegenerative conditions, dermatological conditions (lipoid proteinosis), and infections, namely intrauterine or perinatal (toxoplasmosis, cytomegalovirus, herpes, rubella) [8]. The low levels of calcium and high levels of phosphorous seen in hypoparathyroidism eventually lead to calcification in different organs like the kidneys (which eventually causes nephrocalcinosis and renal insufficiency), the heart (leading to cardiac arrhythmias and congestive heart failure), the eyes (which lead to posterior subcapsular cataracts), and the cerebrum [9].

## Conclusions

Although Fahr’s syndrome is a very rare neurological entity, the possibility should be kept in mind while diagnosing any patient with a seizure disorder, especially in the third to fourth decade. Clinicians should keep in mind that hypoparathyroidism is a common cause of intracranial calcifications and longstanding hypocalcemia. It should also be noted that, if this is caught early and adequately treated with calcium supplementation, it will halt the calcification process and lead to marked clinical improvement.

To conclude, the practise of medicine reaffirms our faith in its basic tenets. In our patient, had a metabolic cause for seizures been identified early in childhood, administration of prolonged duration of anti-epileptics could have been avoided. Possibly, we could have hoped for a better outcome.
